# Association of timing of menarche with depressive symptoms and depression in adolescence: Mendelian randomisation study

**DOI:** 10.1192/bjp.bp.115.168617

**Published:** 2017-01

**Authors:** Maija-Eliina Sequeira, Sarah J. Lewis, Carolina Bonilla, George Davey Smith, Carol Joinson

**Affiliations:** **Maija-Eliina Sequeira**, MSc, **Sarah J. Lewis**, PhD, **Carolina Bonilla**, PhD, **George Davey Smith**, PhD, School of Social and Community Medicine, and MRC Integrative Epidemiology Unit (IEU), University of Bristol, Bristol, UK; **Carol Joinson**, PhD, School of Social and Community Medicine, University of Bristol, Bristol, UK

## Abstract

**Background**

Observational studies report associations between early menarche and higher levels of depressive symptoms and depression. However, no studies have investigated whether this association is causal.

**Aims**

To determine whether earlier menarche is a causal risk factor for depressive symptoms and depression in adolescence.

**Method**

The associations between a genetic score for age at menarche and depressive symptoms at 14, 17 and 19 years, and depression at 18 years, were examined using Mendelian randomisation analysis techniques.

**Results**

Using a genetic risk score to indicate earlier timing of menarche, we found that early menarche is associated with higher levels of depressive symptoms at 14 years (odds ratio per risk allele 1.02, 95% CI 1.005–1.04, *n* = 2404). We did not find an association between the early menarche risk score and depressive symptoms or depression after age 14.

**Conclusions**

Our results provide evidence for a causal effect of age at menarche on depressive symptoms at age 14.

Observational studies have shown that females experience higher lifetime rates of depression^1,2^ and that this difference emerges in mid-puberty and persists throughout adulthood.^2,3^ Findings from the UK Psychiatric Morbidity Survey indicate no further divergence in rates of depression between males and females after adolescence,^4^ suggesting that adolescence is the key developmental period for the emergence of gender differences in depression. It has been suggested that aspects of the pubertal transition may underlie the observed increase in depression in females during adolescence.^5^ Many studies use menarche – the onset of first menstrual bleeding – as a proxy for onset of puberty in females, and associations between early menarche and increased levels of depressive symptoms and depression in adolescence are widely reported. Cross-sectional studies report higher levels of depressive symptoms and depression in girls who have experienced menarche compared with girls who have not yet experienced this transition.^3,6,7^ Many longitudinal studies report that girls with early menarche have higher levels of depressive symptoms and depression in adolescence compared with those with later-onset menarche.^8–15^

It is unclear, however, whether the apparent effect of early menarche on depressive symptoms/depression persists in the longer term. Findings from the Avon Longitudinal Study of Parents and Children (ALSPAC) cohort suggest that by late adolescence (16–18 years), girls with later-onset menarche experience similar levels of depressive symptoms to those with early menarche.^11^ However, limitations of observational studies, including confounding, reverse causality and bias, make findings difficult to interpret. Although randomised controlled trials (RCTs) are lauded as the ‘gold standard’ of epidemiological research methods, it would not be possible to examine the relationship between age at menarche and depression within an RCT design.^16^ Mendelian randomisation analysis has been proposed as a way of determining whether the relationship between an exposure and outcome is causal, by obtaining a non-confounded assessment of the relationship.^17^ Mendelian randomisation exploits Mendel's first and second laws. At a population level, genetic variants (alleles) will not be associated with confounding factors if: (a) the probability that a germ cell with any particular allele contributes to a viable conception is independent of the environment (the principle of segregation), and (b) allele pairs segregate independently of each other (the principle of independent assortment).^18^ This process is analogous to the randomisation of individuals in a clinical trial. Within a Mendelian randomisation study, a genetic variant that is robustly associated with the exposure of interest can be used as an instrumental variable in analyses,^16^ serving as a genetic proxy for the exposure (Fig. 1).

**Fig. 1:**
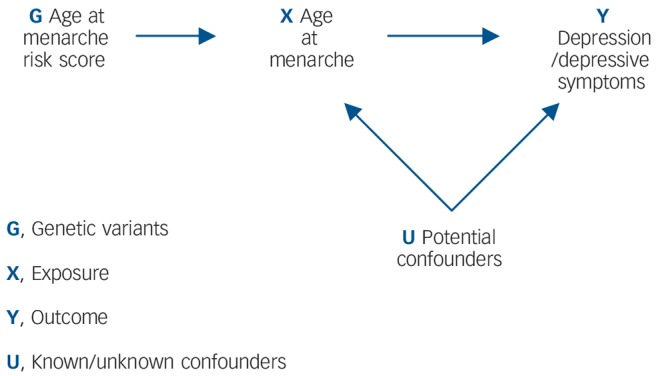
Addressing the causal directions of effect in the association between age at menarche and depressive symptoms/depression, with the use of allelic risk scores and Mendelian randomisation analysis.

Since it can be assumed that confounders of the relationship between the exposure and outcome are not associated with the genetic variants, this approach is particularly useful in instances where traditional epidemiological methods are likely to suffer from confounding. The underlying assumptions of Mendelian randomisation are that:^16,19^ (a) the genotypes are robustly associated with the non-genetic exposure of interest; (b) there is no association between the genotypes and the factors that confound the relationship between the non-genetic exposure and outcome of interest; (c) the genotypes only affect the outcome through the exposure of interest. To our knowledge no previous study has used causal analysis techniques to examine the relationship between early menarche and depression/depressive symptoms. The aim of this study is to exploit Mendelian randomisation analysis to provide a non-confounded assessment of these associations. We specifically examine: (a) whether early onset of menarche is causally associated with depressive symptoms and depression in adolescence; and (b) whether this effect persists into late adolescence.

## Method

### Study sample

**Fig. 2:**
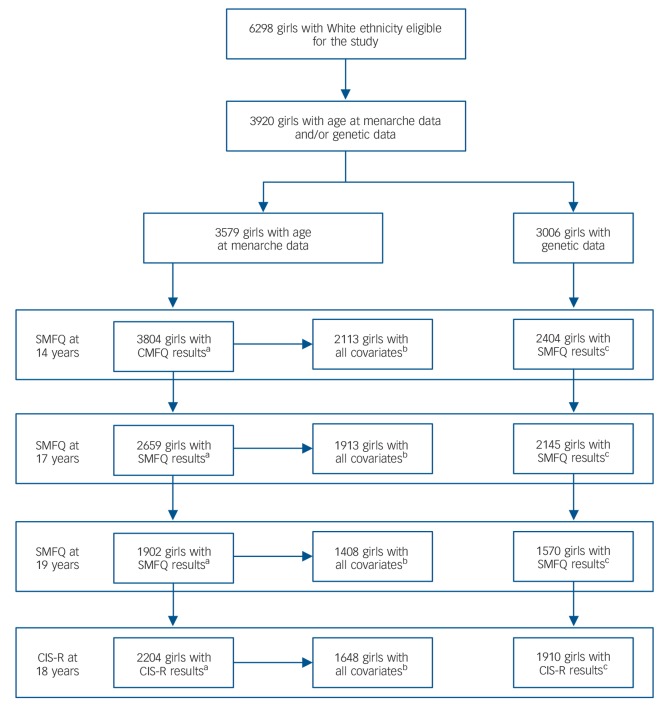
Flow chart showing sample sizes included in observational and Mendelian randomisation analyses. a, Sample sizes for unadjusted observational analyses; b, sample sizes for adjusted observational analyses; c, sample sizes for Mendelian randomisation analyses. SMFQ, Short Moods and Feelings Questionnaire; CIS-R, Clinical Interview Schedule-Revised.

Analyses were carried out on data from ALSPAC, a population-based longitudinal study that recruited pregnant women resident in the Avon area of the UK with an expected delivery date between 1 April 1991 and 31 December 1992.^20,21^ The initial cohort included 14 541 pregnancies and 13 988 children alive at 12 months (*n* = 6762 girls). When the oldest children were 7 years old, attempts were made to recruit children who were eligible for, but did not join, the sample at the initial phase.^22^ In total, 713 new children (*n* = 392 girls) were recruited, resulting in a starting sample for the current study of 7154 (6762+392) girls. Only girls with White ethnicity were eligible for this study (*n* = 6298); of these, 3920 had at least one outcome measure, 3579 had data on age at menarche, and 3006 had genetic data (Fig. 2).

The ALSPAC website contains details of all available data through a fully searchable data dictionary (http://www.bris.ac.uk/alspac/researchers/data-access/data-dictionary/). The study and cohort are described extensively elsewhere^21,22^ with further details available at: http://www.bristol.ac.uk/alspac/. Ethical approval for the study was obtained from the ALSPAC Law and Ethics Committee and the local research ethics committees. Written informed consent was obtained from all study participants.

### Exposure

#### Age at menarche

Age at menarche was derived from a series of nine postal questionnaires pertaining to pubertal development, sent approximately yearly from age 8 to 17 years. The questionnaires asked ‘Has your daughter started her menstrual periods yet?’ and, if yes: ‘How old was your daughter when she had her first period?’ Answers were given in years and months. These data were supplemented by questionnaires administered to girls at two research clinics attended at 12 years 10 months and 13 years 10 months asking ‘Have you started your periods yet?’ and, if yes: ‘When did you have your first period?’ The first-reported age at onset was used, as these results are least likely to be affected by recall bias.

### Outcomes

#### Depressive symptoms

The Short Moods and Feelings Questionnaire (SMFQ) enquires about the occurrence of depressive symptoms over the past 2 weeks, with response categories ‘True’, ‘Sometimes’, or ‘Not at all’ for 13 items. The SMFQ has been validated as a tool for assessing depressive symptoms in adolescence^23^ and distinguishes children with depression from those who are not depressed in general population samples.^24^ This study uses SMFQ data collected at mean ages 13 years 10 months, 16 years 8 months, and 18 years 8 months, hereafter referred to as 14, 17 and 19 years.

At age 14, participants completed the SMFQ at a research clinic whereas at 17 and 19 years it was included in questionnaires mailed to participants. We dichotomised the SMFQ scale and defined high levels of depressive symptoms as a score of 11 or above. At this cut-off the SMFQ has been found to have high sensitivity, specificity and negative predictive power, and high discriminatory ability for an ICD-10 diagnosis of depression at 18 years.^23^ This cut-off has been used in ALSPAC^7,8,25^ and other^26,27^ studies previously.

#### Depression

Participants completed a self-administered computerised version of the Clinical Interview Schedule-Revised (CIS-R) at a research clinic (mean age 17 years 7 months, hereafter 18 years).^28,29^ The CIS-R measures affective and anxiety disorders in the past week and has been widely used to diagnose depression according to ICD-10 criteria. It is standardised, has been validated in adolescents, and is equally reliable whether self-administered or conducted by a trained interviewer.^28,29^ The depression diagnosis examined in this study is ‘any ICD-10 diagnosis of depression’, referring to any mild, moderate or severe depressive episode.

### Genotyping and imputation

The children in ALSPAC were genotyped using the Illumina HumanHap550 quad chip (Illumina, Inc., San Diego, California, USA) by the Wellcome Trust Sanger Institute, Cambridge, UK and the Laboratory Corporation of America, Burlington, USA. The resulting raw genome-wide data were subjected to standard quality control methods. Individuals were excluded on the basis of gender mismatches; minimal (<0.325) or excessive heterozygosity (>0.345); disproportionate levels of individual missingness (>3%); cryptic relatedness measured as proportion of identity by descent (IBD>0.1) and insufficient sample replication (IBD<0.8).

The remaining individuals were assessed for evidence of population stratification by multidimensional scaling analysis and compared with Hapmap II (release 22) European descent (CEU), Han Chinese (CHB), Japanese (JPT) and Yoruba (YRI) reference populations; all individuals with non-European ancestry were removed. Single nucleotide polymorphisms (SNPs) with a minor allele frequency of <1%, a call rate of <95% or evidence for violations of Hardy–Weinberg equilibrium (*P*<5 × 10^−7^) were removed. Genotypic data were subsequently imputed using Markov Chain Haplotyping software^30^ and phased haplotype data from the Thousand Genomes Project (2010–2011 data freeze) that included 1092 samples of mixed ethnicity who had had singleton and monomorphic sites removed.

### Covariates

A range of covariates pertaining to the study child, parents and socioeconomic level were identified from the literature as potential confounders of the relationship between age at menarche and depressive symptoms/diagnosis.^7,8,11,27,31–36^ We included measures of body mass index (BMI) from 9 and 15 years, proxies for pre- and post-menarche BMI, since they are potentially associated with age at menarche through different mechanisms.^32–34^ BMI was derived from height and weight measurements taken during research clinics at 9 and 15 years. Where these were missing, BMI was obtained from height and weight reported in questionnaires administered at 9 and 15 years (correlation coefficient 0.846).^11^ The number of older siblings was obtained from an antenatal questionnaire and categorised into (a) none, (b) one, or (c) two or more.

Maternal education was reported during the antenatal period and defined as (a) certificate of secondary education or vocational qualifications, (b) O-level qualifications, or (c) A-level and higher qualifications. Maternal age at birth of the study child was derived from questionnaires administered to the mother. Antenatal and postnatal depression were derived from maternal questionnaires; women self-reported antenatal depression at 18 and 32 weeks' gestation, and also completed a series of questions between 8 weeks' and 61 months' post-birth, from which an Edinburgh Postnatal Depression Score (EPDS)^37^ was calculated. The EPDS was dichotomised at a cut-off of 12/13, the standard cut-off used to indicate probable depressive disorder.^38^ We derived father absence from a series of questionnaires completed by the mother. Since there is evidence that family breakdown in the first 5 years may have a greater influence on subsequent timing of menarche,^31,35^ the variable was categorised to (a) father not absent (b) father absent before child was age 5, or (c) father absent when child was between 5 and 10 years.

Mother's and partner's occupations were reported in the antenatal period and social class was derived using the 1991 Office of Population Censuses and Surveys (OPCS) job codes: (a) professional occupations; (b) managerial and technical occupations; (c) skilled occupations (non-manual); (d) skilled occupations (manual); (e) partly-skilled occupations; or (f) unskilled occupations. Financial problems were reported by the mother in a number of questionnaires between 8 weeks and 61 months and we derived a variable that reflected any experience of financial problems, and the effect on the family as perceived by the mother.^35^ This was categorised as: (a) no financial problems; (b) any reporting of financial problems, with a small effect; or (c) any reporting of financial problems, with a large effect. Home ownership was reported in questionnaires completed between 8 weeks' gestation and 122 months' post-birth. We included the first reported answer, and the variable was dichotomised as living in (a) owned or mortgaged property, or (b) rented property.

### Statistical analysis

All analyses were carried out in Stata 13 MP2.

#### Descriptive and observational analyses

For continuous variables we calculated means and standard deviations and explored the distribution of each variable. We carried out regression analyses between the risk score and potential confounders of the exposure–outcome relationship in order to determine whether they were associated, which would violate assumption 2 of Mendelian randomisation.

We carried out unadjusted logistic regression between reported age at menarche and depressive symptoms/depression using the ‘logistic’ command. We then adjusted the analyses for the covariates described above.

#### Deriving the genetic score

A recent genome-wide association study (GWAS) meta-analysis carried out across 57 studies with a total of up to 182 416 women of European descent found 123 signals that are associated with age at menarche.^39^ We extracted these variants from the genetic data available in ALSPAC and tested for deviation from Hardy–Weinberg equilibrium using the Stata command ‘hwsnp’. We excluded two variants (rs17233066 and rs929843) from the score on the basis of low imputation *R*^2^ values (*R*^2^ = 0.36 and 0.02, respectively) and a third (rs16896742) that was neither genotyped nor imputed in the ALSPAC cohort.

We summed the genetic dosages of the remaining 120 variants to create a non-weighted genetic risk score for age at menarche. Genetic dosages range from 0 to 2 at each site and represent the expected number of age-at-menarche-reducing alleles for each individual. We also created a categorical score by dividing the risk score into quartiles, allowing the dose–response relationship to be explored in analyses. We regressed the genetic risk score against age at menarche in order to assess direct associations of the allele score and phenotype (online Table DS1).

#### Mendelian randomisation analyses of genetic score and depressive symptoms/depression

We carried out logistic regression analyses between the risk scores and depressive symptoms/depression using the ‘xi:logistic’ command in Stata. Ten principal components were included to control for confounding by population stratification.^40^ We then carried out instrumental variable analyses of age at menarche and depressive symptoms/depression with the ‘ivreg2’ command, using the continuous risk score as an instrumental variable. We used a structural mean model,^41^ allowing for binary outcomes by using the ‘robust’ option, and again adjusted for all ten principal components. *F*-statistics from the first-stage regression between genetic risk scores and age at menarche were examined to ensure that the instrument is strongly associated with the exposure (using *F*>10 as an arbitrary threshold), reducing the risk of weak instrument bias.^16^ The ‘ivreg2’ command estimates a causal risk difference in the outcome (depressive symptoms/depression) per unit change in the exposure (age at menarche).

#### Sensitivity analyses

ALSPAC was included in the discovery GWAS meta-analysis,^39^ violating the assumption of independence between discovery and study samples that is required for polygenic risk score analyses.^42^ In order to correct for this violation of sample independence we carried out sensitivity analyses in which we removed the contribution of ALSPAC from the effect sizes reported in the GWAS. We recalculated the overall effect under a fixed-effect model after removing the estimate from the ALSPAC study, by subtracting the quantities contributed by this study (effect estimate and weight) from the weighted mean. We then created a new weighted score, in which the effects of ALSPAC were removed, and repeated regression and instrumental variable analyses at age 14 years using this new score. We were also aware that the existence of pleiotropy – when a genetic variant has an effect on outcome independent of its effect on the exposure – would violate assumption 3 of Mendelian randomisation.^17^ We were aware of potential pleiotropic effects of age at menarche SNPs with BMI^43,44^ and thus carried out sensitivity analyses to determine the effect of this.

Ideally we would do this by excluding only those SNPs associated with pre-menarche BMI from the risk score. Excluding SNPs associated with post-pubertal BMI would risk introducing collider bias, since we would essentially stratify analyses by BMI.^45^ In this case, post-pubertal BMI is the collider and the other variables are age at menarche and the confounders of the relationship between BMI and depression. If we exclude post-pubertal BMI SNPs, then we risk inducing an association between age at menarche and these confounders which, in turn, could lead to a spurious association with depression.^46^

Unfortunately, no published research that we are aware of has identified SNPs distinctly associated with ‘pre- and post-menarche’ rather than ‘childhood’ BMI. We were, however, able to carry out some sensitivity analyses by creating three new risk scores that excluded SNPs that were found in: (a) a GWAS of BMI in adulthood;^47^ (b) a GWAS of BMI in childhood;^48^ and (c) either the childhood or adulthood GWAS. We re-ran Mendelian randomisation analyses with these three scores, adjusted for ten principal components as before.

Furthermore, we used Bowden's Mendelian randomisation–Egger regression test^49^ in order to assess the potential violation of assumption 3 of Mendelian randomisation (i.e. no direct effect of the genetic variants on the outcome). This provides a valid test of directional or unbalanced pleiotropy and a consistent estimate of the true causal effect.^49^ The slope of the Mendelian randomisation–Egger regression represents this true causal effect, and the intercept provides evidence of pleiotropy if different from zero.

## Results

### Descriptive and observational analyses

The sample sizes used for analyses at each time point vary as a result of availability of data but can be observed in Fig. 2.

The mean age at menarche was 12 years 8 months (approximately 152 months), and this was similar across all groups used in analyses (online Table DS2). The mean SMFQ score and variability in the score increased with age (online Table DS1). At 14 years, the proportion of girls with depressive symptoms was highest among early maturers and lowest among late maturers. By 19, the differences between the groups appear to be negligible (online Table DS3).

All included SNPs were in Hardy–Weinberg equilibrium. The genetic risk score had a normal distribution around a mean value of 121.39 age-at-menarche-reducing alleles (online Fig. DS1) with little change across the different samples (online Table DS4).

There was a strong negative association of the score with age at menarche whereby a one-allele increase in the continuous risk score was associated with a decrease in age at menarche of 0.42 months or approximately 11 days (95% CI −0.49 to −0.35, *P* = 1.410^−28^) (Table 1). Girls in the first, second, third and fourth quartiles of the categorical risk score experienced menarche at mean ages of 12 years and: 11 months; 9 months; 8 months; and 6 months respectively.

**Table 1:** Results of linear regression analyses of the continuous genetic risk score against the continuous age at menarche variable and confounders

		Genetic risk score	
Variable	*n*	Estimate (95% CI)	*P*
Age at menarche (months)	2772	−0.42 (−0.49 to −0.35)	1.410^−28^

Body mass index at age 9	2705	0.04 (0.02 to 0.05)	2.010^−5^

Body mass index at age 15	2122	0.05 (0.03 to 0.07)	2.010^−5^

Maternal antenatal depression	2590	0.01 (−0.01 to 0.02)	0.34

Maternal postnatal depression	2684	−0.002 (−0.02 to 0.01)	0.80

Maternal age	2709	−0.02 (−0.06 to 0.02)	0.30

Maternal education	2745	−0.001 (−0.01 to 0.00)	0.81

Social class	2645	1.010^−14^ (−0.01 to 0.01)	0.98

Financial problems	2704	−0.003 (−0.01 to 0.00)	0.20

Home ownership	2922	−0.004 (−0.02 to 0.01)	0.56

Father absence	2747	0.002 (0.00 to 0.01)	0.15

Siblings, *n*	2741	1.010^−14^ (0.00 to 0.00)	0.85

#### Depressive symptoms

Continuous age at menarche (months): after adjusting for confounders we found strong evidence that a 1-month increase in age at menarche was associated with 1% lower odds of depressive symptoms at 14 years (95% CI 0.98–0.995, *P* = 0.003), however, this association attenuated at 17 (95% CI 0.99–1.003, *P* = 0.20) and 19 (95% CI 0.99–1.01, *P* = 0.85) years (online Table DS5).

Categorical age at menarche (early/normative/late onset): adjusted analyses suggest a protective effect of late menarche on depressive symptoms at both 14 and 17 years, whereby girls who experienced menarche late had respectively 32% (95% CI 0.50–0.92, *P* = 0.01) and 28% (95% CI 0.54–0.95, *P* = 0.02) lower odds of depressive symptoms compared with normative onset menarche (online Table DS5).

#### Depression

There was insufficient evidence to suggest that age at menarche was associated with diagnosed depression at 18 years in adjusted analyses when using either the categorical (95% CI 0.46–1.07, *P* = 0.10) or the continuous (95% CI 0.98–1.004, *P* = 0.16) risk scores (online Table DS5).

### Analyses using the genetic risk scores

#### Mendelian randomisation analyses

There was strong evidence for an association between both the continuous and categorical risk scores and higher levels of depressive symptoms at 14 years. On average a one-allele increase in the risk score was associated with a 2% increase in the odds of depressive symptoms at 14 years (95% CI 1.005–1.04, *P* = 0.01) (Table 2). Girls in the fourth quartile of the risk score experienced menarche on average 5 months earlier than girls in the first quartile and had 74% higher odds of depressive symptoms at 14 years (95 CI 1.26–2.40, *P* = 0.001).

**Table 2:** Mendelian randomisation analyses: odds ratios for depressive symptoms at ages 14, 17 and 19 years (SMFQ⩾11), and for depression diagnosis consistent with ICD-10 criteria at age 18, by continuous and categorical risk scores

	OR (95% CI)	*P*
*Continuous risk score*		
SMFQ at 14 (*n* = 2404)	1.02 (1.005–1.04)	0.01
SMFQ at 17 (*n* = 2145)	1.002 (0.99–1.02)	0.78
SMFQ at 19 (*n* = 1570)	1.001 (0.98–1.02)	0.88
CISR at 18 (*n* = 1910)	1.004 (0.98–1.03)	0.73

*Categorical risk score*		
Depressive symptoms (SMFQ) at 14 (*n* = 2404)		
2nd Quartile	1.62 (1.18–2.25)	0.003
3rd Quartile	1.38 (0.99–1.92)	0.05
4th Quartile	1.74 (1.26–2.40)	0.001
Trend	1.15 (1.04–1.27)	0.005
Depressive symptoms (SMFQ) at 17 (*n* = 2145)		
2nd Quartile	0.95 (0.71–1.26)	0.73
3rd Quartile	0.90 (0.68–1.21)	0.49
4th Quartile	1.08 (0.82–1.43)	0.59
Trend	1.02 (0.93–1.12)	0.68
Depressive symptoms (SMFQ) at 19 (*n* = 1570)		
2nd Quartile	0.74 (0.52–1.04)	0.08
3rd Quartile	0.81 (0.58–1.12)	0.20
4th Quartile	1.06 (0.76–1.46)	0.74
Trend	1.02 (0.92–1.14)	0.70
Depression (CIS-R) at 18 (*n* = 1910)		
2nd Quartile	0.84 (0.55–1.28)	0.41
3rd Quartile	0.83 (0.54–1.26)	0.37
4th Quartile	1.14 (0.76–1.70)	0.53
Trend	1.04 (0.91–1.19)	0.57

SMFQ, Short Moods and Feelings Questionnaire; CIS-R, Clinical Interview Schedule-Revised.

There was insufficient evidence to suggest that the association between the categorical or continuous risk scores and depressive symptoms persisted at 17 or 19 years, nor was there evidence of an association with diagnosed depression at 18 years (Table 2).

#### Instrumental variable analyses

A first-stage *F*-statistic of 114.9 (*P*<0.0001) indicates a very strong instrument that explains 4.93% of variability in age at menarche, confirming that assumption 1 of Mendelian randomisation is fulfilled. At 14 years we obtained a result of a causal risk difference of 0.0062, meaning that there will be an additional 6 girls per 1000 (or 62 per 10 000) with depressive symptoms, for each month of earlier menarche.

#### Sensitivity analyses

In total 13 of the 123 SNPs in the discovery GWAS failed to reach genome-wide significance (510^−8^) without the contribution of ALSPAC, although 12 of these showed suggestive associations with age at menarche (*P*<510^−5^). Alongside the 3 SNPs previously excluded from the score, this left us with a weighted score composed of 107 SNPs. Results with this score were consistent with those obtained with the original unweighted score and with the weighted score that included the effects of ALSPAC (Table 3).

**Table 3:** Sensitivity analyses: coefficients for depressive symptoms at age 14 years (SMFQ⩾11) by unweighted, weighted (including ALSPAC) and weighted (excluding ALSPAC) genetic risk scores

	Coefficient (95% CI)	OR (95% CI)^a^	*P*
Unweighted score			
Association of score with depressive symptoms at 14		1.02 (1.005 to 1.04)	0.01
Association of score with age at menarche	−0.42 (−0.49 to −0.35)^b^		<0.001
Instrumental variable analysis	0.006 (0.001 to 0.01)^c^		0.02

Weighted score (including ALSPAC)			
Association of score with depressive symptoms at 14		1.04 (1.01 to 1.07)	0.02
Association of score with age at menarche	−0.86 (−1.00 to −0.72)^b^		<0.001
Instrumental variable analysis	0.006 (0.001 to 0.01)^c^		0.03

Weighted score (excluding ALSPAC)			
Association of score with depressive symptoms at 14		1.03 (0.998 to 1.07)	0.06
Association of score with age at menarche	−0.74 (−0.89 to −0.59)^b^		<0.001
Instrumental variable analysis	0.006 (−0.001 to 0.01)^c^		0.07

SMFQ, Short Moods and Feelings Questionnaire; ALSPAC, Avon Longitudinal Study of Parents and Children.

a. OR per unit increase in score.

b. Change in age at menarche in months per unit increase in score.

c. Risk difference per month decrease in age at menarche.

Of the 123 SNPs associated with age at menarche, five were robustly associated with adult BMI (rs543874, rs3101336, rs10938397, rs7138803 and rs12446632), and one with childhood BMI (rs8050136). After excluding these SNPs from the respective scores and re-running analyses, findings were consistent with analyses undertaken using the complete risk score (online Table DS6).

The Mendelian randomisation–Egger test results show no evidence of directional or unbalanced pleiotropy, with an intercept value of −0.01 (95% CI −0.04 to 0.02, *P* = 0.65), and a pooled estimate of the causal effect of a month decrease in age at menarche on depressive symptoms at 14 years of an OR = 1.03 (95% CI −0.98 to 1.08, *P* = 0.27).

## Discussion

### Main findings

We found strong evidence for a causal effect of age at menarche on depressive symptoms in mid-adolescence (14 years). This effect did not persist into late adolescence (17–19 years), nor was there evidence for a causal effect of age at menarche on depression at 18 years. To the best of our knowledge this is the first time that Mendelian randomisation techniques have been used to examine whether the widely reported association between age at menarche and depressive symptoms/depression is causal. These techniques allow us to confirm that the association observed is not solely as a result of confounding.

### Strengths and limitations

The major strengths of this study are the longitudinal multiwave design of ALSPAC, repeated measures of depressive symptoms and depression from mid- to late-adolescence, and the availability of genetic data. Since ALSPAC was included in the GWAS that identified the genetic variants used in this analysis, our original results might overestimate the effects of the genetic variants in relation to age of menarche (overfitting). However, we attempted to reduce this likelihood by: (a) using an unweighted score and therefore not taking the potentially biased effect estimates into account in analyses; and (b) repeating analyses with a weighted score that excluded the effects of ALSPAC from the original GWAS. This process of ‘de-meta-analysis’ does, however, introduce error into the new effect estimates and therefore our weighted score – although unbiased by the exclusion of ALSPAC – is itself an imperfect instrument.

Testing for the potential pleiotropic effects of the score on the estimates of the effect of age at menarche on depressive symptoms using Mendelian randomisation–Egger shows no evidence of pleiotropy and a similarly inverse association as that obtained with instrumental variable regression. However, Mendelian randomisation–Egger is quite a conservative test and provides a causal estimate with large standard errors.

A further potential limitation of our study is that it may be underpowered to detect a causal effect at older ages, since the sample size for the adjusted analyses was reduced by approximately a third between 14 and 19 years (Fig. 2) due to sample attrition. However, previous analyses in ALSPAC used continuous depressive symptom latent traits across samples with varying degrees of missing data, and drew robust conclusions across samples with complete and partial depressive symptoms.^11^ Furthermore, the consistent mean SNP score and standard deviation (online Table DS4) suggest that the study populations were genetically similar.

### Potential explanations for our findings

This study provides increased understanding of the link between menarche and depressive symptoms/depression. With regard to our specific research questions: first, we find evidence for a causal association between early menarche and increased levels of depressive symptoms in mid-adolescence. Second, we do not find evidence that the adverse effect of early menarche on depressive symptoms persists into late adolescence. Hormonal, neurocognitive and psychosocial factors could explain why girls with early menarche experience increased levels of depressive symptoms in mid-adolescence. Puberty is associated with an overall increase in hormone levels, and also with dramatic fluctuations in the levels of oestrogen circulating in the body.^1^ The body's systems must rapidly adapt to higher and fluctuating levels of hormones that may influence mood. For instance, the hypothalamic–pituitary–adrenal (HPA) axis has to mature rapidly and become sensitised to new hormonal feedback mechanisms.^1^ Dysregulation of the HPA axis has been linked to a susceptibility to depression in women,^50^ and therefore onset of menarche may increase depressive symptoms through this mechanism. Furthermore, these hormonal and neurocognitive changes occur around the time that girls are exposed to a range of new biological and psychosocial stressors,^6^ making them particularly vulnerable to dysregulation during this sensitive transition period.

The psychosocial ‘off-time’ and ‘early-timing’ hypotheses^51^ have been explored in studies examining the link between age at menarche and depressive symptoms/depression.^8,10,15^ They suggest that girls who experience menarche out of sync with (‘off-time’) or significantly before (‘early-timing’) their peers are more likely to develop depressive symptoms/depression. Inconsistency between levels of biological and cognitive maturation, as well as feelings of being ‘different’ to one's peers, may induce psychological distress and depressive symptoms.^6^ The early-timing hypothesis further explains the higher levels of depression and depressive symptoms in early-maturing girls by suggesting that they are particularly ill-equipped to deal with the psychosocial consequences of puberty because of their relative cognitive immaturity at the time of menarche.^51^

Our findings suggest that the mood-related changes linked to onset of menarche are transient since in late adolescence (i.e. when all girls have experienced the menarcheal transition) there were no differences in the level of depressive symptoms according to timing of menarche. This is consistent with previous findings from a number of prospective studies, including an earlier study with the ALSPAC cohort,^11^ suggesting that the associations between timing of menarche and depressive symptoms/depression dissipate in late adolescence.^9,13,14,52^

Higher levels of depressive symptoms/depression observed in mid-adolescence could be due to the fact that girls with earlier menarche are experiencing this rise earlier than their peers. The apparent differences in depressive symptoms/depression in earlier-*v.* later-maturing girls may then dissipate as a result of two concurrent processes: ‘catch-up’ by girls with later-onset menarche as they experience the rise in level of depressive symptoms associated with the menarcheal transition, and ‘recovery’ (i.e. reduced levels of depressive symptoms) in girls who experienced early menarche.^13^

There are suggestions of catch-up by later maturers in our study (online Table DS3), and recovery had been explored previously. A prospective study that mapped the trajectories of depression before and after puberty found that rates of depression in girls followed an inverse U-shaped curve over age, falling steadily after reaching a peak in late adolescence (16–17 years) and continuing to fall until at least 23 years.^52^ This raises the question of whether the girls with late-onset menarche in our study might still have high transitional levels of depressive symptoms in late adolescence, and might eventually recover to a lower level of depressive symptoms than girls who experienced earlier menarche. Further collection of data on depressive symptoms in the ALSPAC cohort will enable investigation of this research question.

Studies examining depression risk by age at menarche in the longer term have had mixed outcomes, with some indicating higher adult levels of depression among women who had late menarche,^12^ and others among those with early menarche.^53^ Further studies are needed that include measures of depression outcomes throughout adulthood to examine whether there are long-term adverse effects of early-onset menarche.

### Implications and extensions of this research

Our findings have important implications for understanding the aetiology of depression and depressive symptoms. We have found evidence for a causal effect of early-onset menarche on depressive symptoms in mid-adolescence, supporting results found in observational studies. For healthcare practitioners, there should be awareness that onset of menarche is associated with a rise in depressive symptoms, and that girls who experience early menarche will experience this rise in depressive symptoms at an earlier age.

Together with the replication of our findings in other cohorts, we suggest that further Mendelian randomisation studies may help to uncover the mechanisms and pathways through which menarche influences the risk of depressive symptoms/depression. Such studies may highlight potentially modifiable targets for intervention.^11^ The consistent results obtained from sensitivity analyses suggest that the effects of menarche on depressive symptoms/depression do not act solely through menarche-related changes in BMI. The publication of a GWAS that robustly identifies the SNPs associated with pre- and post-pubertal BMI would enable clarification of the pathways linking menarche, depressive symptoms/depression and BMI.
